# Preclinical magnetic resonance imaging and spectroscopy in the fields of radiological technology, medical physics, and radiology

**DOI:** 10.1007/s12194-024-00785-y

**Published:** 2024-02-14

**Authors:** Shigeyoshi Saito, Junpei Ueda

**Affiliations:** 1 Department of Medical Physics and Engineering, Area of Medical Imaging Technology and Science, Division of Health Sciences, Osaka University Graduate School of Medicine, Suita, Osaka 560-0871 Japan; 2 Department of Advanced Medical Technologies, National Cerebral and Cardiovascular Center Research Institute, Suita, 564-8565 Japan

**Keywords:** Magnetic resonance imaging, Preclinical MRI, Small-animal high-field MRI equipment, Permanent magnet animal MRI equipment

## Abstract

Magnetic resonance imaging (MRI) is an indispensable diagnostic imaging technique used in the clinical setting. MRI is advantageous over X-ray and computed tomography (CT), because the contrast provided depends on differences in the density of various organ tissues. In addition to MRI systems in hospitals, more than 100 systems are used for research purposes in Japan in various fields, including basic scientific research, molecular and clinical investigations, and life science research, such as drug discovery, veterinary medicine, and food testing. For many years, additional preclinical imaging studies have been conducted in basic research in the fields of radiation technology, medical physics, and radiology. The preclinical MRI research includes studies using small-bore and whole-body MRI systems. In this review, we focus on the animal study using small-bore MRI systems as “preclinical MRI”. The preclinical MRI can be used to elucidate the pathophysiology of diseases and for translational research. This review will provide an overview of previous preclinical MRI studies such as brain, heart, and liver disease assessments. Also, we provide an overview of the utility of preclinical MRI studies in radiological physics and technology.

## Introduction

Recently, several imaging modalities, including magnetic resonance imaging (MRI) [1–3], radiography, computed tomography (CT) [4–9], positron emission tomography (PET) [10, 11], single-photon emission computed tomography (SPECT) [12, 13], electron microscopy, autoradiography, optical imaging, and ultrasound (US) [14–17], have been applied in biomedical, genetic, toxicologic, cancer, cardiology, and pharmacological research worldwide. These are all noninvasive imaging modalities with proven clinical applications that have been extended to in vivo and ex vivo research specimens as small as mice, rats, and marmosets [9, 18, 19]. The ability to perform noninvasive repeated imaging in the same animal enables us to study the longitudinal progression of diseases or treatments using these modalities. Moreover, the localization of morphological and functional changes in the organ systems of small animals provides information on embryonic development [20], genetic mutations [21], potential therapeutic treatments [22, 23], environmental factors, and their impacts [24–26]. Preclinical research, also known as translational research, plays a critical role in the development of clinical practice. Using animal models and other laboratory techniques such as MRI and CT to investigate the mechanisms of diseases and develop potential treatments, researchers can develop new diagnostic tools, therapies, and preventive measures that can improve human health. In recent decades, the importance of preclinical imaging has been shown by the increasing number of commercial imaging systems available for most modalities. Preclinical imaging studies have been conducted for many years in basic research in the fields of radiological technology, medical physics, and radiology. The preclinical MRI research includes studies using small-bore and whole-body MRI systems. In this review, “preclinical MRI” mainly define for the animal study of small-bore MRI. While, human whole-body MRI systems are also available for preclinical use. However, we will only address some of them in whole-body MRI systems in this review.

### Preclinical MRI platform in Japan

MRI is an essential diagnostic imaging technique in clinical practice. Its advantage over radiography and CT lies in its ability to provide a contrast that depends on density differences in organ tissues. The number of MRI machine density in Japan is the largest in the world, exceeding 50 per million population, and MRI plays an important role in diagnostic imaging, similar to radiography, CT, and US. In addition to hospital MRI systems, Japan has more than 100 “Research MRI equipment,” which is used in numerous fields, including basic research, molecular and clinical elucidation of the mechanism, and life science research, such as drug discovery, veterinary medicine, and food testing. Over the years, considerable efforts have been made to apply clinical MRI imaging techniques to small-animal experiments. However, only a limited number of researchers and technicians can access preclinical MRIs in Japan. One possible reason is that preclinical MRI systems can be very expensive to purchase and maintain. In particular, the recent depreciation of the yen and exchange rate fluctuations have become a major problem for purchases of medical and scientific equipment from outside Japan. In addition, the lack of specialized personnel is another reason for the low number of users of the preclinical MRI equipment. The acquisition of MRI measurement techniques and animal experimental techniques is essential for the use of preclinical MRI. Such human resources are still scarce in Japan. Saito and his collaborators launched a new platform for MRI research, supported by the Advanced Research Equipment Platform within the Project for Promoting Public Utilization of Advanced Research Infrastructure within the Ministry of Education, Culture, Sports, Science, and Technology (MEXT), Japan (https://mripf.jp/). By virtually connecting such as remote operating, and data share using crowd system for research MRI equipment in different facilities across Japan, they aimed to create a research environment that could be shared by researchers across the country, thus allowing them access to the most advanced MRI equipment in the country. To date, the platform has published more than 80 paper results (https://mripf.jp/archive.html) [23, 27–43].

Several press releases have been issued to date on the achievements of this platform. For example, the researchers identified that homozygous truncating mutations in the gene encoding Bcl-2-associated athanogene (BAG) co-chaperone 5 (BAG5) caused inherited dilated cardiomyopathy in patients. Then, preclinical cardiac MRI imaging and echocardiography revealed left-ventricular dilatation and reduced systolic function in the hearts of BAG5 knock in mice compared to those of wild-type mice without apparent histological abnormalities [28].

In addition, task-free functional connectivity in animal models such as rat and mice provide an experimental framework to examine connectivity phenomena under controlled conditions and allows for comparisons with data modalities collected under invasive or terminal procedures in preclinical neuroscience study using small-bore MRI. Currently, animal acquisitions are performed with varying protocols and analyses that hamper result comparison and integration in worldwide. StandardRat is a consensus rat functional magnetic resonance imaging acquisition protocol tested across 20 centers which include National Institutes for Quantum Science and Technology (QST) in Japan. This protocol and processing pipeline described here is openly shared with the neuroimaging community to promote interoperability and cooperation toward tackling the most important challenges in neuroscience [32]. Thus, their platform will continue to actively disseminate the latest preclinical and translational research using preclinical MRI.

### Preclinical MRI equipment

The preclinical MRI research includes studies using small-bore and whole-body MRI systems [44]. This paper focused on studies using small-bore MRI systems for several reasons. First, small-bore MRI systems are specifically designed for preclinical research and are optimized for imaging small animals such as mice and rats. This allows for higher spatial resolution and sensitivity compared to whole-body MRI systems, which are designed for larger animals and humans. Second, small-bore MRI systems are more widely used in preclinical research than whole-body MRI systems, and therefore, there is a larger body of literature available for review and analysis. Finally, the researchers have chosen to focus on small-bore MRI systems because they may be more accessible to researchers, especially those in academic or small-scale research settings who may not have access to whole-body MRI systems. Indeed, preclinical MRI is still not accessible for researchers and technicians in the field of radiological physics and technology. Therefore, this review provides an opportunity for more researchers to become familiar with preclinical MRI. It is important to note that the choice of MRI system used in preclinical research depends on the research question and the animal model being studied. Whole-body MRI systems may be more appropriate for certain types of research, such as studies on large animals or on whole-body physiology. Ultimately, the choice of MRI system depends on the specific needs and goals of the research project.

**Fig. 1:**
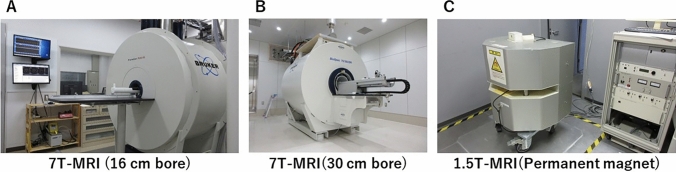
**A** Preclinical 7 T MRI (16 cm small bore, helium autonomy 150 days with nitrogen compressor system). **B** Preclinical 7 T MRI (30 cm wide bore, zero helium boil-off technology, nitrogen-free auto-bed system). **C** Preclinical 1.5 T MRI (open permanent magnet gradient, with the motor-driven automatic positioning system AutoPac™)

High-field superconductor and permanent magnet MRI systems for preclinical and small-animal use are available from several manufacturers (Bruker, Agilent, MRSolution, Mediso, Japan REDOX, among others) and differ from those used clinically. The number of preclinical and small-animal high-field MRI systems and permanent magnet animal MRI systems installed at facilities in Japan currently exceeds 100, with static magnetic field strengths of 1.0 T, 1.5 T, 4.7 T, 7 T, and 9.4 T, and the highest magnetic field of 11.7 T in Japan. The author administered two 7 T MRI and 1.5 T permanent magnet machines at Osaka University and the National Cerebral and Cardiovascular Center Research Institute (Fig. 1). Figure 1A shows that preclinical 7 T MRI (16 cm small bore, for mice and rats) consists of 150 days of helium autonomy with a nitrogen compressor system. Figure 1B shows that preclinical 7 T MRI (30 cm wide bore, for mice, rats, and rabbits) consists of a zero helium boil-off technology, nitrogen-free, and auto-bed system (AutoPac™). The motor-driven automatic positioning system simplifies animal handling and allows streamlined calibrated positioning for effortless and reproducible precision during routine scanning. Permanent magnet systems have a limited number of sequences that can be used for imaging; however, superconducting systems can be used for fast imaging sequence such as echo-planar imaging (Fig. 1C).

In preclinical small-bore high filed MRI, diffusion tensor imaging (DTI) [45], magnetic resonance spectroscopy (MRS) [30, 46], magnetic resonance angiography (MRA) [47], arterial spin labeling (ASL) [48], susceptibility-weighted imaging (SWI) [49], and other imaging techniques that use these sequences are available. ASL, SWI, chemical exchange saturation transfer imaging (CEST) [23, 29, 31, 50], Q-space imaging (QSI) [51], and relaxation time measurements of T1-, T2-, and T1rho-mapping [27] are available and can be used for most imaging methods applied in clinical practice.

**Fig. 2:**
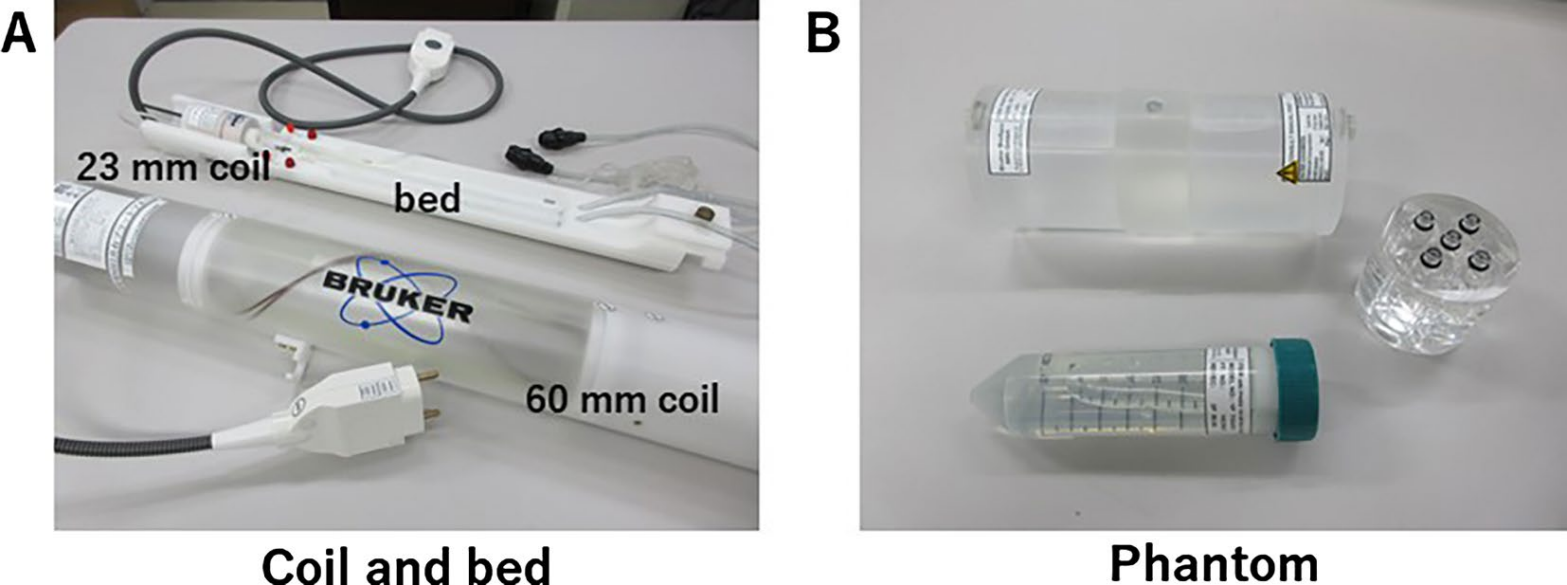
**A** Coils and bed (mice cradle bed with 23 mm brain coil and a 60 mm rat body coil). **B** Phantom (top: quality control for 60 mm coil, right: hand-made diluted series phantom, and bottom: quality control for 40 mm)

Furthermore, the number of available sequences increases every year due to regular software updates. Preclinical equipment typically uses a dedicated animal bed and an attached MRI coil (Fig. 2A). Phantoms were also prepared for the maintenance of these coils (Fig. 2B). Some imaging methods can be modified more extensively than clinical systems, including respiratory synchronization, electrocardiography synchronization, and phased-array coils in the case of higher end systems. Additionally, the system is compatible with ^31^P and ^13^C coils, making it possible to perform multinuclear MRS [52, 53].

### Previous reports of preclinical MRI research in the journal of radiological physics and technology

Several studies on radiological physics and technology (RPT) have reported the use of MRI in animals. The first report on RPT was published by Yamamoto et al. in 2009 [54]. Their study attempted to evaluate the feasibility of developing a whole-body 3 T MRI system for small animals. The quality of various images, including anatomic T1W, T2W, time-of-flight magnetic resonance angiography (TOF-MRA), and dynamic susceptibility contrast images, was assessed in rats [54]. This small-animal imaging system with clinical scanners is important to directly address clinical questions and identify the origins of signal changes, including various disease conditions, in a clinical setting. In 2011, a second study by Nagata et al. on RPT for animal MRI research evaluated the Mn content in various organs of rats and the change in Mn content after glucose stimulation in vivo and in vitro using MRI and polarized Zeeman atomic absorption spectrophotometry, respectively, to investigate the feasibility of Mn-enhanced MRI for estimating β-cell mass and function in the pancreas [55]. They used a permanent MRI system for small animals. MRI studies were performed using a 1.5 T MRI system for animal experiments (MRmini SA, DS Pharma Biomedical Co., Ltd, Osaka, Japan), in which a solenoid coil of 38.5 mm diameter was used as the radiofrequency coil. The new MRI equipment (MR VivoLVA) was transferred to the Japanese redox system.

**Fig. 3:**
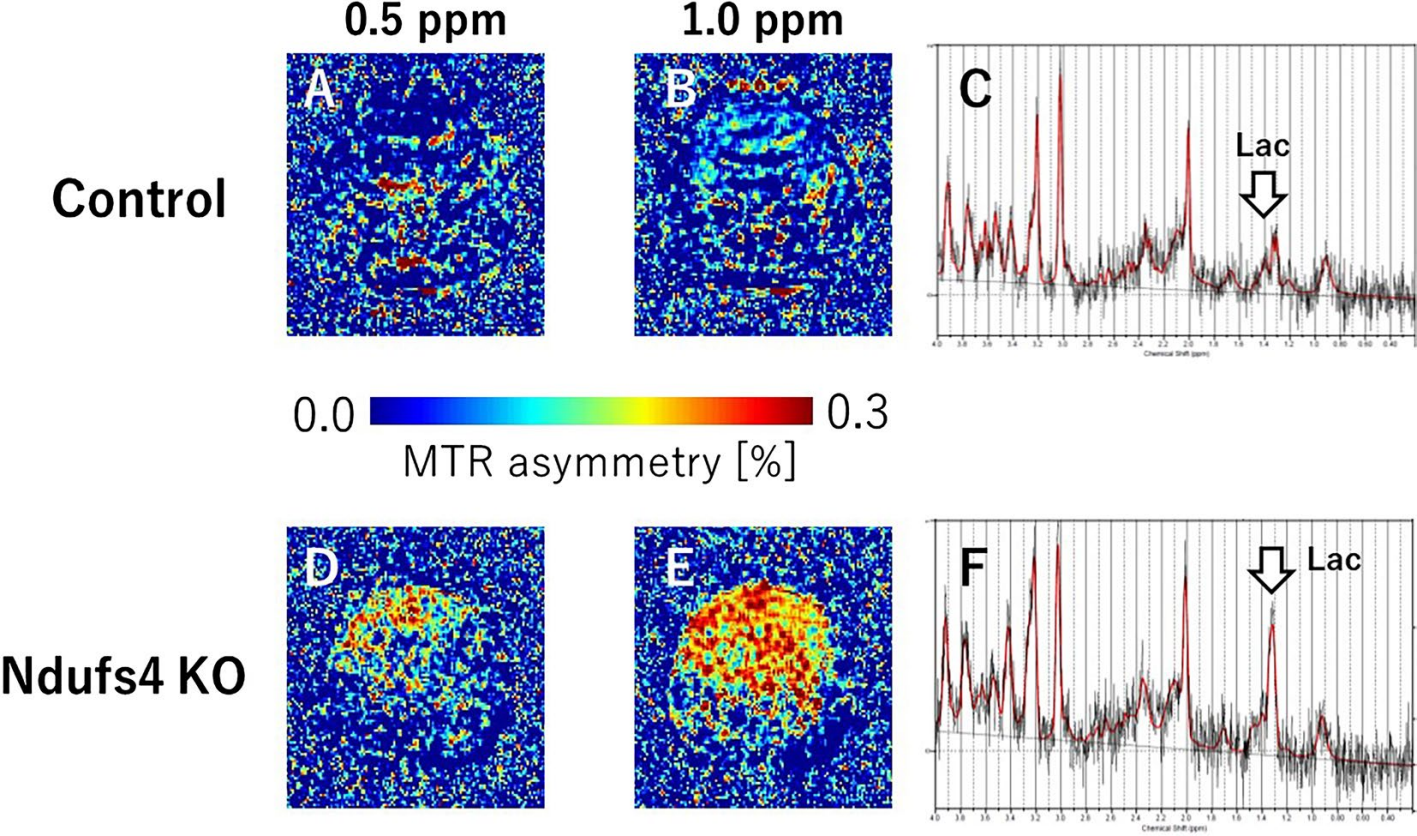
Lactate-CEST imaging of mouse brains. The MTR asymmetry maps of 0.5 ppm and 1.0 ppm of the KO mice (**A**, **B**) are higher than those of the control WT mice (**D**, **E**). Lactate levels in KO mice (**C**) are significantly higher than those of control mice (**F**)

Their preclinical MRI study used a 7 T MRI Bruker preclinical system. Their study aimed to use CEST; Fig. 3A, B, D and E) and MRS (Fig. 3C, F) with 7 T MRI for the early detection of intracerebral lactate in a mitochondrial disease model without brain lesions [56]. CEST imaging has emerged as a powerful tool for detecting biomarkers such as pH, glucose, and protein concentration in vivo. CEST MRI has been used to investigate a wide range of diseases, including cancer, stroke, and neurodegenerative disorders, and has shown promise as a noninvasive method for disease diagnosis and monitoring. Saito et al. used Ndufs4-knockout (KO) mice as a Leigh syndrome model [46, 57] and wild-type (WT) mice as controls. Brain MRI and ^1^H-MRS were performed. CEST has the potential to be a sensitive tool for detecting intracerebral lactate in some diseases and clarifying related physiological functions. Another study detected intracerebral lactate in a mitochondrial disease model using CEST and MRS with 7 T MRI [56]. Therefore, preclinical MRI studies in RPT journals were initially reported using clinical machines for animal experiments, followed by permanent magnet and superconducting animal machines.

### Anesthesia and physiological monitoring for small-animal MRI research

The most important factor in preclinical and small-animal MRI examinations is the degree to which animal movement can be controlled. In clinical MRI, the subject's movement during imaging must be minimized as much as possible, and the scan timing and movement of the organs must be accurately monitored and synchronized with the imaging and factored into the reconstruction process. An overview of the anesthetics used in the MRI of mice and rats and biological monitoring during MRI is described.

**Fig. 4:**
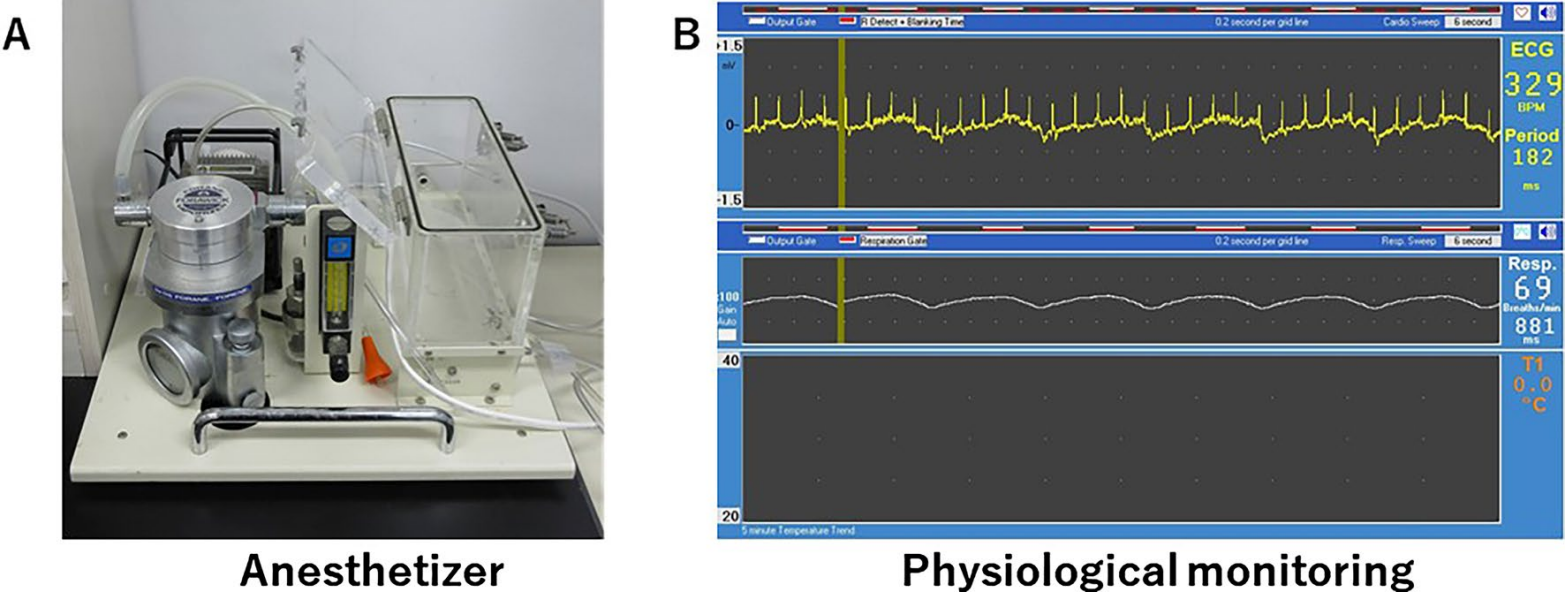
**A** Anesthetizer and **B** biological monitoring (ECG, respiratory, and body temperature)

Laboratory animals are anesthetized for various purposes, many of which aim to reduce pain. The Animal Welfare Law stipulates that animal experiments should be conducted using methods that cause minimal pain. Compared to injectable anesthetics, inhalation anesthetics induce safe general anesthesia that can be used for short or long periods of time, allowing easy adjustment of the depth of anesthesia, and the animal awakens in a short time. However, in isoflurane-anesthetized rats, even a short hypoxic episode can have long-lasting effects on cerebrovascular regulation [58]. In recent years, inhalation anesthesia machines specially designed for small animals have become available from several manufacturers and are relatively easy to use. Isoflurane and sevoflurane are used most frequently in MRI. In their laboratory, several types of isoflurane inhalation anesthesia machines are used: one for imaging (Fig. 4A), which can be operated from the side of the console, and the other for induction before imaging. Furthermore, some anesthetics are used, with attention paid to differences in animal strains and sexes. Mice tend to be hypothermic compared to rats, and their body temperature is affected by thermal insulation, room temperature, and other factors. Therefore, it is necessary to consider variations in the depth of anesthesia, even at the same dose, depending on age, concomitant drugs, body condition, and manipulated genes. In their laboratory, body temperature was maintained at 37 °C by circulating 55 °C warm water in a tube placed near the abdomen during imaging.

The physiological monitoring of rodents and marmosets during MRI is generally performed using non-blood-intensive techniques. Monitoring equipment and instruments should be MRI-compatible to avoid noise and artifacts in the image, as well as for safety reasons. In the imaging of mice and rats, respiration and body temperature were measured, and electrocardiograms were used (Fig. 4B). In marmoset imaging, pulse rate and blood oxygen saturation (SpO_2_) were measured simultaneously. Marmoset imaging is performed mainly using an MRI-compatible animal biomonitoring device (SAI), and SpO_2_ measurements are performed using pulse oximetry. Accurate and precise monitoring of body temperature, respiration, and electrocardiography can reduce the risk of death in experimental animals.

### Preclinical MRI for brain and neuroimaging

**Fig. 5:**
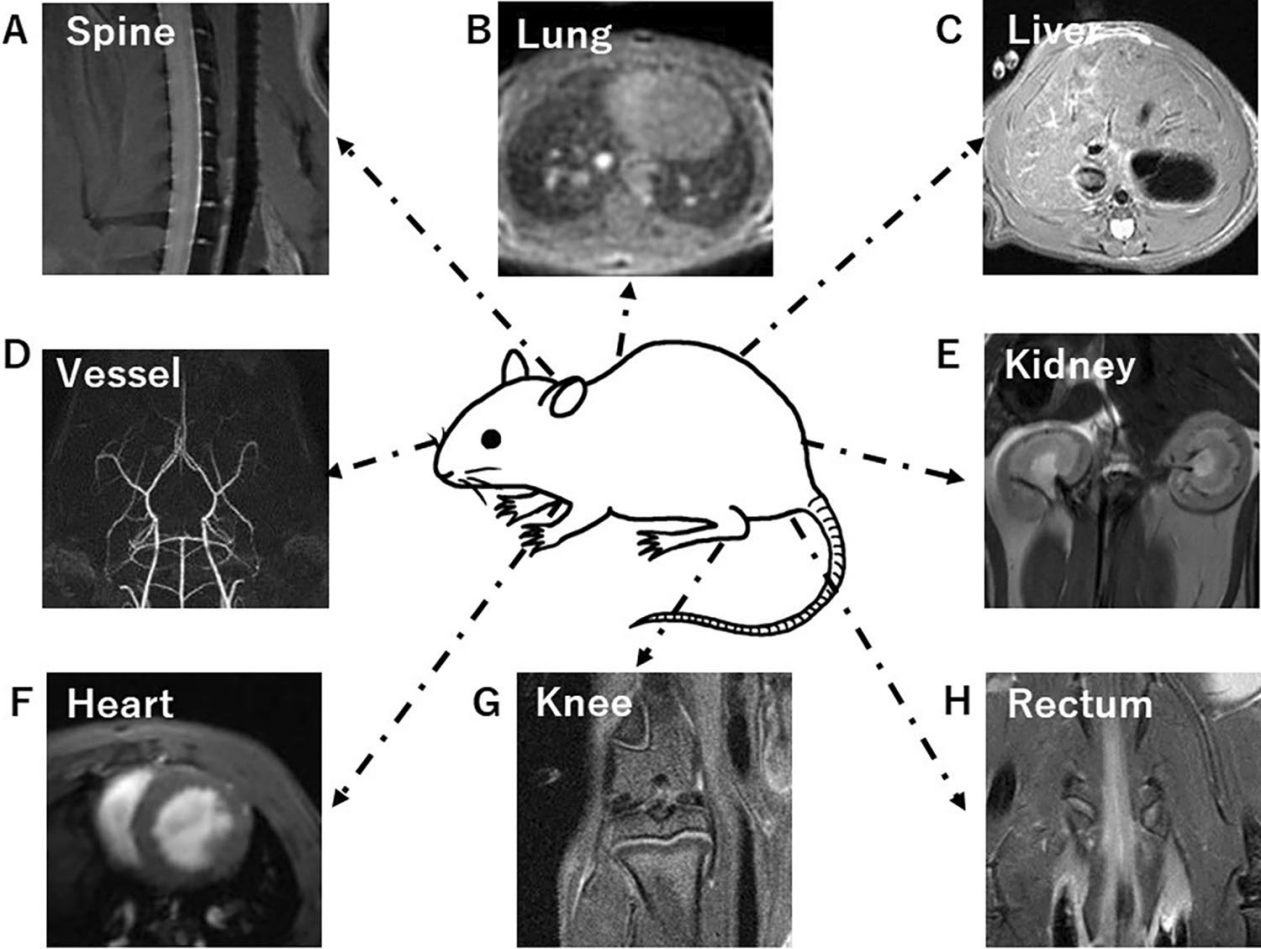
**A** Mouse spine, **B** mouse lung, **C** Rat liver, **D** mouse MR angiography, **E** rat kidney, **F** Rat heart cine imaging, **G** rat knee, and **H** mouse rectum

Preclinical MRI targets include the spine, lung, liver, arteries, kidney, heart, knee, and rectum of rodents (Fig. 5A–H) and small primates, as well as tissue and biological samples with a resolution of ≤ 50 µm. This is a major distinction from clinical imaging, as rodents, such as mice and rats, require imaging under anesthesia. Although imaging while awake is sometimes used to measure brain function in marmosets, it is technically difficult, because the coil shape for prone position must be made, the keep of the prone position is difficult, and MRI noise training for subject animals is required.

**Fig. 6:**
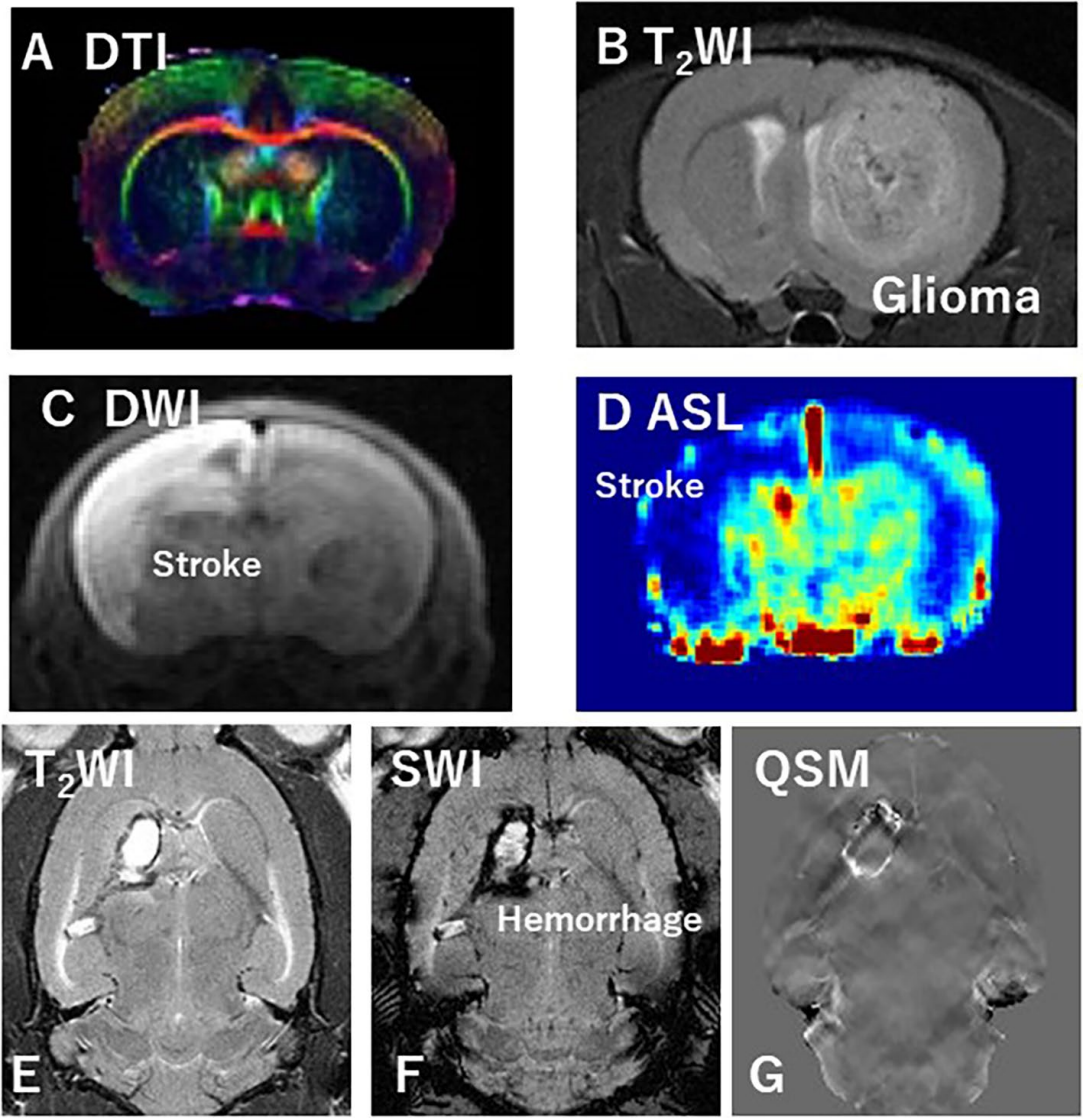
**A** DTI of the rat brain, **B** rat glioma; **C** DWI of the rat brain stroke model, **D** ASL of the rat brain stroke model, **E**, **F**, and **G** T2WI, SWI, and QSM of a rat brain hemorrhage model, respectively

Neuroimaging in preclinical MRI studies is the most widely used modality, as well as in clinical MRI research. Neuroimaging techniques, such as DTI, fMRI, and MRS, provide a noninvasive evaluation of brain structure and activity and have been used to determine the possible mechanisms of cognitive aging in humans. Studies using DTI (Fig. 6A), fMRI, and MRS in animal models have been applied as an integrative set of techniques to bridge localized cellular and molecular phenomena and broader in vivo brain alterations in disease models.

Onishi et al. [23]and Tanoue et al. [59] established orthotopic brain tumor mouse and rat models and performed multiparametric MRI analysis. A brain tumor model was created by transplanting C6 rat glioma cells directly into the brain (Fig. 6B). They then used the C6 glioma rat model to evaluate tumor changes caused by chemotherapy at different doses of temozolomide (TMZ) in terms of quantitative values measured by neurite orientation dispersion and density imaging (NODDI) and amide proton transfer imaging using a 7 T MRI system. Microstructural changes caused by TMZ-induced tumor growth inhibition can be evaluated using multiparametric MRI analysis [23].

**Fig. 7:**
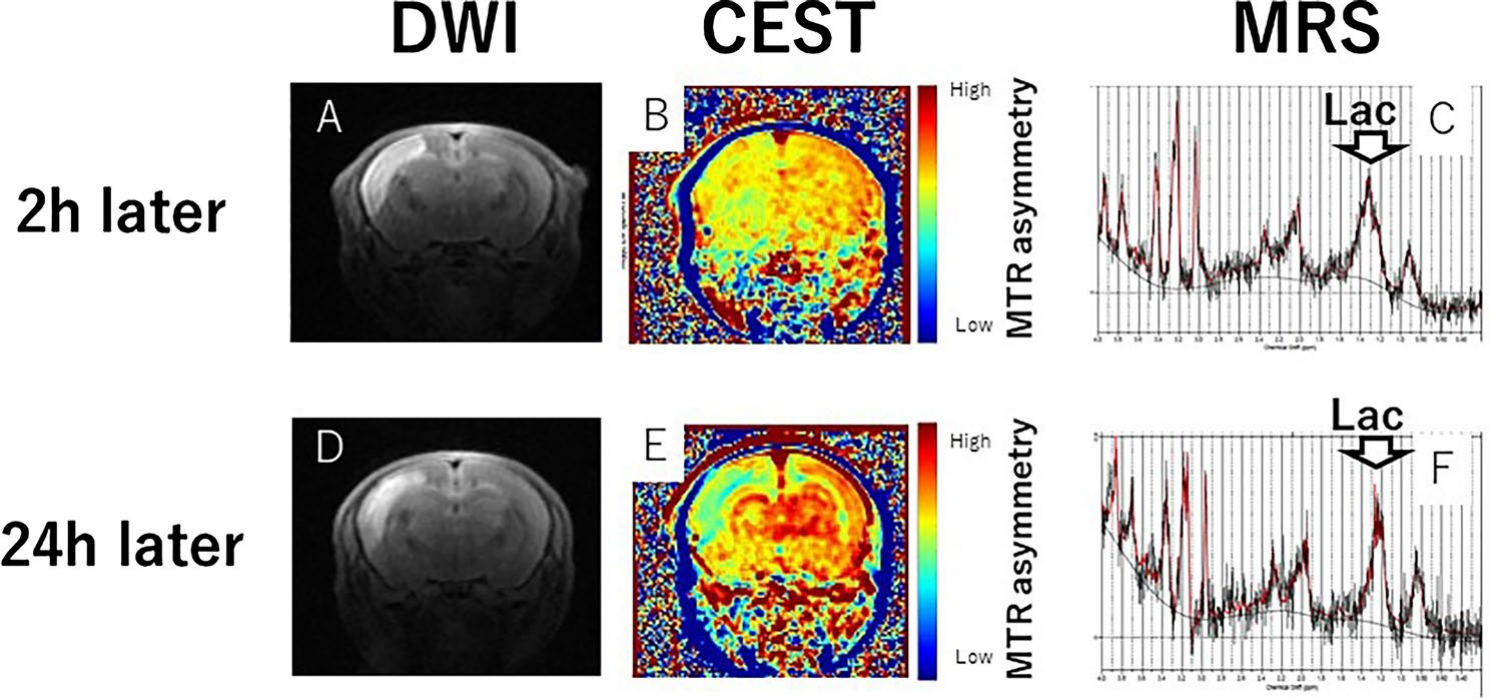
**A** DWI of ischemic rat brain as 2 h later, **B** CEST image of ischemic rat brain as 2 h later, **C** MRS of ischemic rat brain as 2 h later, **D** DWI of ischemic rat brain as 24 h later, **E** CEST image of ischemic rat brain as 24 h later, and **F** MRS of ischemic rat brain as 24 h later

Stroke is the second most common cause of death and the third most common cause of disability-adjusted life years lost worldwide [60]. Ischemic stroke is caused by poor blood flow in the brain. Therefore, a highly reproducible animal model that can verify new MRI findings for cerebral infarction is necessary. Studies on in utero cerebral infarction models are underway. Hypoxic-ischemic encephalopathy (HIE) is caused by perinatal asphyxia and causes serious impairments, including mental retardation, epilepsy, and cerebral palsy. Ohki et al. evaluated the effects of CEST on ischemic regions in HIE compared to DWI and MRS using 7 T MRI (Fig. 6C and Fig. 7A–F) [32]. The CEST MTR maps did not correspond to the hyperintense areas of DWI at 0 to 2 and 24 h after the onset of HIE (Fig. 7B and E). Changes in the multi-offset CEST signal may be primarily related to brain metabolites and pH alterations, such as those caused by lactate, after the onset of HIE (Fig. 7C and F). Furthermore, they evaluated the utility of NODDI to longitudinally assess the severity of HIE. Using NODDI, quantitative maps for the intracellular volume fraction (ICVF), orientation dispersion index (ODI), and isotropic volume fraction (ISO) are generated. Their NODDI results revealed reduced FA at 168 h and elevated ICVF, ISO, or ODI at varying time points from 1 to 168 h in severe vs. mild HIE. This suggests that NODDI is useful for identifying the severity of the HIE model in the early phase. NODDI is a potential method to determine the severity of neonatal HIE in rats.

Stroke can be classified as ischemic or hemorrhagic. As mentioned above, ischemic stroke is caused by a loss of blood supply to an area of the brain and is a common form of stroke. Hemorrhagic stroke is caused by bleeding in the brain due to ruptured blood vessels. Hemorrhagic stroke can be further divided into intracerebral hemorrhage (ICH) and subarachnoid hemorrhage (SAH). They created a rat model of ICH and compared quantitative susceptibility mapping (QSM) with the conventional MRI during the longitudinal evaluation of ICH. To establish this model, collagenase was injected into the right striatum of Wistar rats. QSM and conventional MRI were performed at 0, 1, 7, and 28 days after surgery using 7 T MRI. The susceptibility, normalized signal value, and area of the hemorrhage site were compared during the image analysis (Fig. 6E, F and G). In this preclinical study using a rat model of ICH, temporal evaluation of ICH using QSM suggested the possibility of detecting asymmetric iron deposition in relation to normal areas of the brain.

Therefore, neuroimaging in preclinical MRI studies of diseases affecting the central nervous system has a multitude of uses and areas of applied research similar to clinical MRI research. In the future, neuroimaging in preclinical MRI studies will be more widely used, as well as in clinical MRI research.

### Preclinical MRI for heart imaging

Preclinical MRI is a powerful tool for noninvasive imaging of the heart in animal models. It can provide high-resolution, detailed images of the heart and surrounding structures, allowing researchers to study cardiac function, morphology, and tissue composition. Moreover, some of the advantages of preclinical MRI for heart imaging include noninvasive, high resolution, and multimodality.

MRI does not require any invasive procedures, making it a safe and convenient option for repeated imaging of animals. Also, MRI can provide high-resolution images of the heart and surrounding structures, allowing for detailed analysis of cardiac function, morphology, and tissue composition. For assessment of cardiac function, MRI can be used to assess various aspects of cardiac function, such as cardiac output, left-ventricular ejection fraction, and myocardial strain [16, 17, 33, 34, 61] (Fig. 5F) Furthermore, MRI can be used to characterize various aspects of cardiac tissue, such as fibrosis, inflammation, and lipid deposition for characterization of cardiac tissue.

**Fig. 8:**
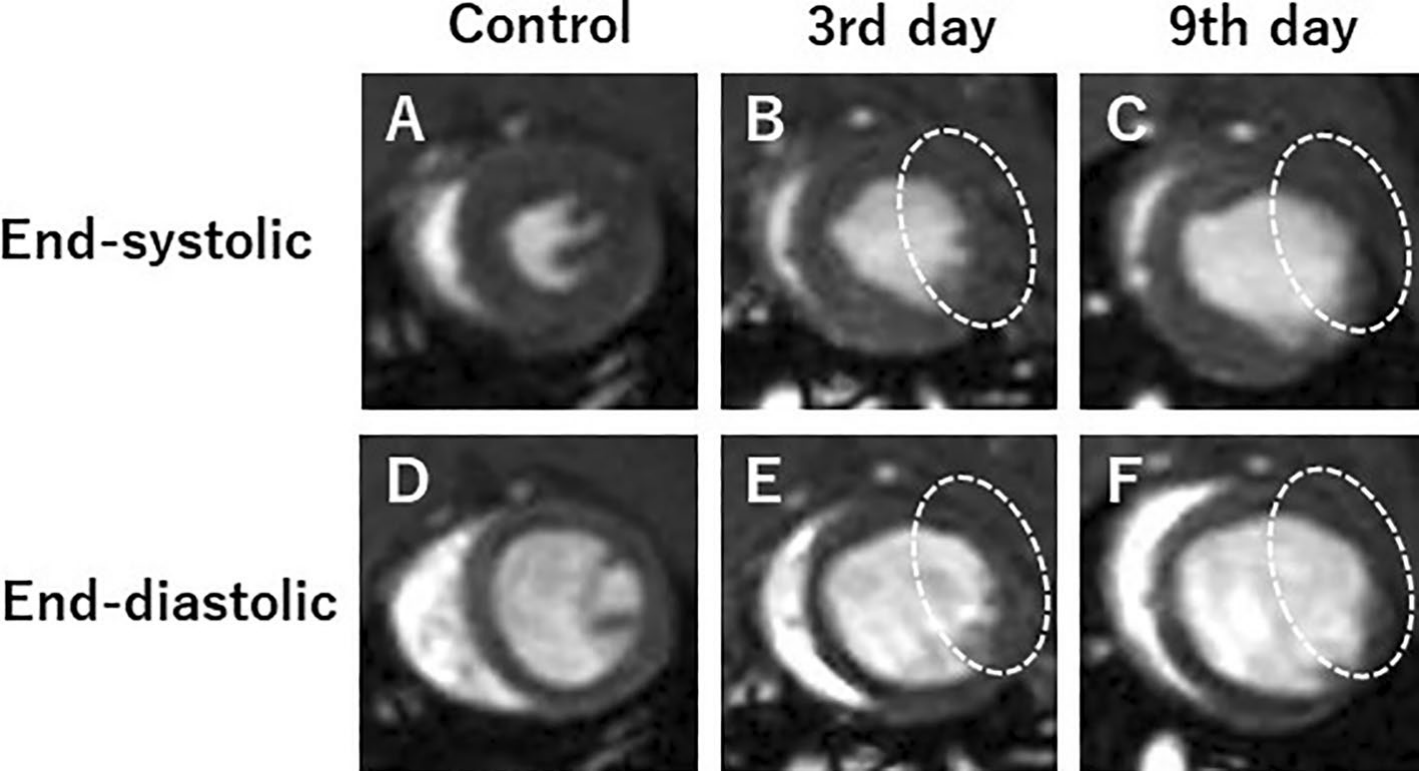
Representative short-axis view cine magnetic resonance images. Control (**A**, **D**), 3 days after the onset of myocardial infarction (**B**, **E**), and 9 days after the onset of myocardial infarction (**C**, **F**). End-systolic phase of the rat heart (**A**, **B**, **C**) and end-diastolic phase of the rat heart (**D**, **E**, **F**). White dotted circles: infarcted area (cited from Tomography 2023, 9(2), 871–882, Fig. 3)

**Fig. 9:**
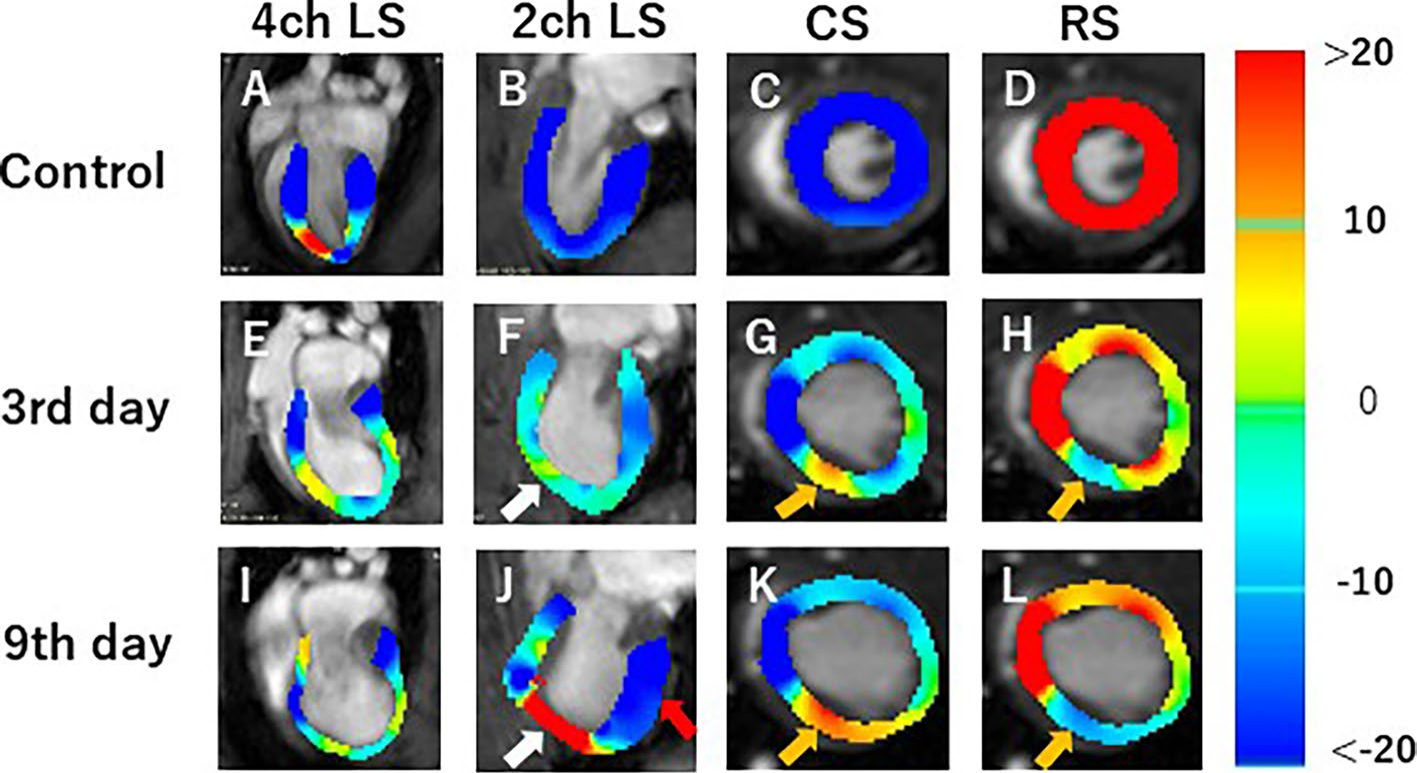
Strain-encoded functional magnetic resonance imaging of the end-systolic left ventricle. 4ch LS, 4ch view longitudinal strain; 2ch LS, 2ch view longitudinal strain; CS, short-axis view circumferential strain; RS, short-axis view radial strain. **A**, **E**, **I** LS in the long-axis 4ch view. **B**, **F**, **J** LS in the long-axis 2ch view. **C**, **G**, **K** CS in the short-axis view. **D**, **H**, **L** RS in the short-axis view. **A**, **B**, **C**, **E** Control, **E**, **F**, **G**, **H** 3 days after the onset of myocardial infarction, and **I**, **J**, **K**, **L** 9 days after the onset of myocardial infarction. The color bar shows the scale of the strain based on the end-diastolic left ventricle, with maximum contraction shown in red and minimum contraction in blue. White arrows: infarcted area. Red arrow: contracting area. Yellow arrows: reduced functionality (cited from Tomography 2023, 9(2), 871–882, Fig. 6)

In Japan, around 200,000 deaths attributed to cardiac diseases reported annually, most of which comprise myocardial infarction (MI). Cardiovascular magnetic resonance (CMR) imaging has recently attracted attention in the evaluation of MI and cardiac function and myocardial tissue-level characteristics. Onishi et al. [33] performed that CMR of MI model rats was acquired on days 3 and 9 after MI using preclinical 7 T MRI, and cardiac function and myocardial strain values were calculated (Fig. 8 and Fig. 9). Rat control cine images and images on days 3 and 9 were evaluated by measuring the left-ventricular ejection fraction (LVEF) and three strain values in the circumferential (CS), radial (RS), and longitudinal directions (LS). In this study, CS decreased significantly 3 days after MI, but there was no difference between images on days 3 and 9. Also, both 2-chamber (2ch) and 4-chamber (4ch) LS values were significantly decreased 3 days after MI. Myocardial strain analysis is, therefore, useful for assessing the pathophysiology and myocardial tissue strain of MI. Overall, preclinical MRI has the potential to greatly advance their understanding of cardiac physiology and pathophysiology.

### Preclinical MRI for liver imaging

Preclinical MRI for liver imaging in animal models can obtain high-resolution imaging of the liver and can provide information on the structure, function, and metabolism of the liver [27, 62–64] (Fig. 5C). One of the advantages of preclinical MRI is its ability to perform dynamic imaging, which can track the uptake and distribution of contrast agents in the liver [64]. This can be particularly useful in the study of liver function and disease, as contrast agents can provide information on liver perfusion, blood flow, and hepatocyte function. In addition, preclinical MRI can be used to study liver pathology, including the development and progression of liver disease, such as fibrosis and cirrhosis. MRI can also be used to monitor the response to therapy in animal models of liver disease, providing valuable information on the efficacy of potential treatments. Overall, preclinical MRI can provide valuable insights into liver function and disease.

**Fig. 10:**
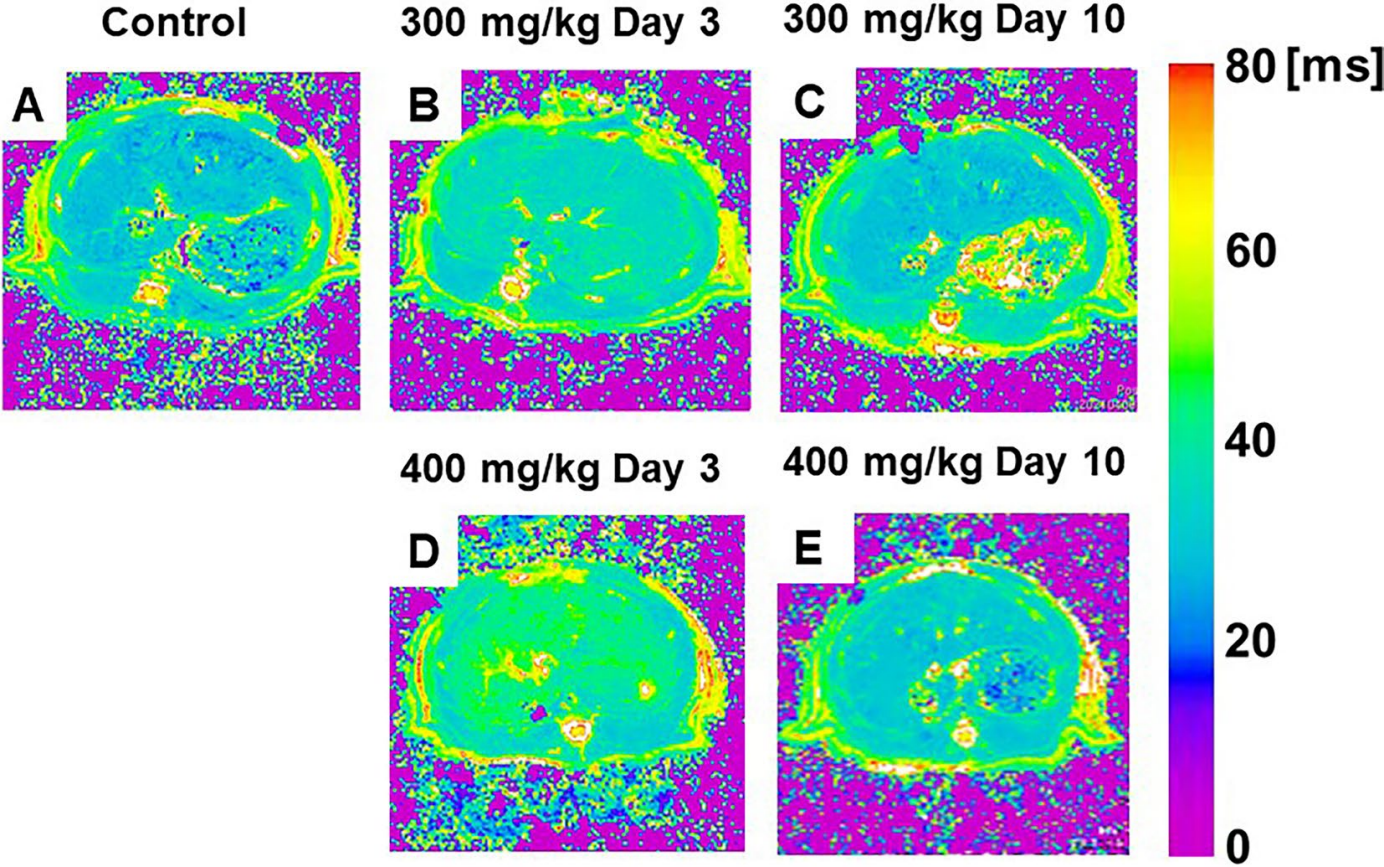
**A**–**E** Color-coded magnetic resonance liver T1rho maps showing representative results in the control (**A**), 300 mg/kg TAA injection (**B**, **C**), and 400 mg/kg TAA injection **D**, **E** models. *TAA* thioacetamide (cited from Metabolites. 2022, Apr 27; 12(5):396, Fig. 1)

**Fig. 11:**
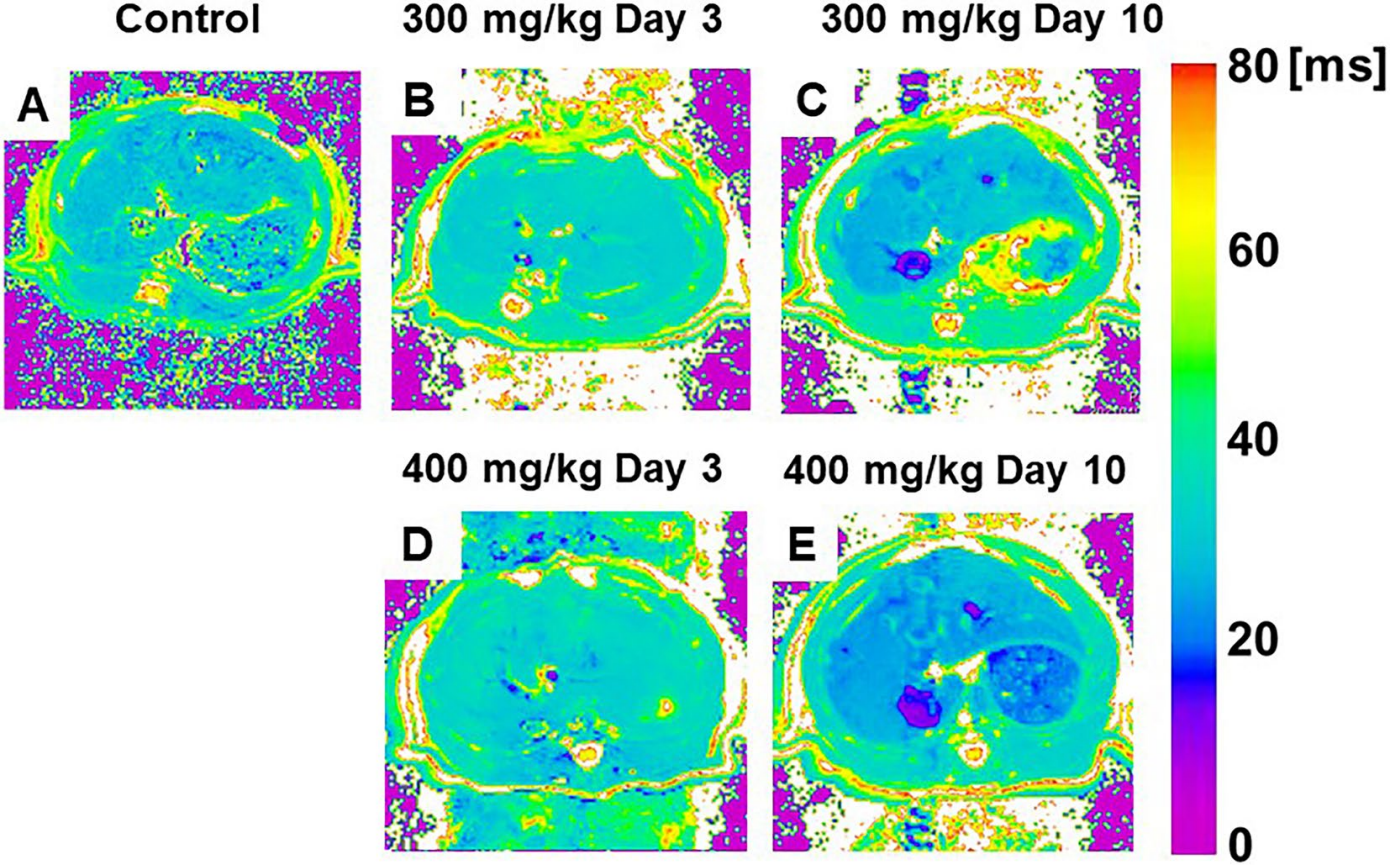
**A**–**E** Color-coded magnetic resonance liver T2 maps showing examples from animals of the control (**A**), 300 mg/kg TAA injection (**B**, **C**), and 400 mg/kg TAA injection (**D**, **E**) groups. *TAA* thioacetamide (cited from Metabolites. 2022, Apr 27; 12(5):396, Fig. 2)

Arihara et al. [27] and Saito et al. [30] measured the relaxation times such as T1rho and T2 values the liver of acute liver inflammation model administered carbon tetrachloride (CCl4) or a thioacetamide (TAA) after dispensed, and they compared and examined whether each relaxation time can be used for detect acute liver inflammation. The T1rho value changed significantly compared to the T2 value, and a continuous change was observed even after 6 days CCl4 administration. While, TAA injection study, liver T1rho and T2 relaxation times significantly increased 3 days after TAA injection compared to pre-injection (Fig. 10 and Fig. 11). Comparing the two TAA models, the high-dose group showed more prolonged T1rho relaxation times than the low-dose group. Three days after TAA administration, the T1rho relaxation time was significantly increased by 45% in the high-dose group in comparison to 29% in the low-dose group (Fig. 10). In T2 maps, in the high-dose group, this parameter was also significantly increased on day 3, and this increase recovered by day 10 after TAA administration (Fig. 11). Thus, relaxation maps of liver may use for diagnosing an acute liver inflammation. In addition, Saito et al. have assessed the liver function in some liver injury and disease models using a dynamic contrast-enhanced MRI with Gd-EOB-DTPA [64–66]. The DCE-MRI with Gd-EOB-DTPA is useful for evaluating some hepatic injury and disease models same as human examinations.

### The challenges for further development of preclinical MRI

There are several challenges that must be addressed to further develop preclinical MRI research in the future. Preclinical MRI systems and infrastructure can be expensive to acquire and maintain, which can limit their accessibility for researchers. This is particularly true for high-field MRI systems such 7 T-, 9.4 T-MRI, which are often the most powerful and high resolution but also the costliest. Also, MRI systems must be compatible with a wide range of animal models, including various sizes, species, and strains. This requires careful design and engineering of the imaging equipment and software. To ensure comparability and reproducibility of results, it is important to establish standardized protocols for preclinical MRI data acquisition and analysis. Despite these challenges, preclinical MRI research has already led to important advances in our understanding of disease mechanisms and potential therapies. As technology continues to advance and new imaging techniques are developed, it is likely that preclinical MRI research will continue to play a key role in advancing medical science in the future.

## Conclusion

Although preclinical imaging systems have been installed in many universities and research facilities, their recognition remains low. Preclinical imaging studies have been conducted for many years in basic research in the fields of radiation technology, medical physics, and radiology. In addition to RPT, we believe that both societies have great potential in terms of the technology and knowledge accumulated by the Japanese Society of Radiological Technology and JSMP, as well as in terms of providing human resources. There remains much potential for applied research, including fusion research in radiotherapy, such as MRI-LINAC, and nuclear medicine, such as PET-MRI. We believe that preclinical MRI research holds great promise in the future of this field.
